# Development and evaluation of an interferon gamma assay for the diagnosis of tuberculosis in red deer experimentally infected with *Mycobacterium bovis*

**DOI:** 10.1186/s12917-017-1262-6

**Published:** 2017-11-16

**Authors:** María Ángeles Risalde, Jobin Thomas, Iker Sevilla, Miriam Serrano, Jose Antonio Ortíz, Joseba Garrido, Mercedes Domínguez, Lucas Domínguez, Christian Gortázar, Jose Francisco Ruíz-Fons

**Affiliations:** 1 SaBio (Health and Biotechnology), Instituto de Investigación en Recursos Cinegéticos IREC (CSIC-UCLM), Ciudad Real, Spain; 2 Unidad de Enfermedades Infecciosas, Instituto Maimonides de Investigación Biomédica de Córdoba (IMIBIC), Córdoba, Spain; 3 Indian Council of Agricultural Research (ICAR), New Delhi, India; 4 NEIKER-Tecnalia, Animal Health Department, Derio, Bizkaia Spain; 5 Medianilla Red Deer Genetics, Benalup, Cádiz Spain; 6 Servicio de Inmunología, Centro Nacional de Microbiología, Instituto de Salud Carlos III, Majadahonda, Madrid Spain; 7 VISAVET Health Surveillance Centre. Complutense University of Madrid, Madrid, Spain

**Keywords:** Animal tuberculosis, Cellular immune response, Farmed *Cervus elaphus*, In vivo test, *Mycobacterium tuberculosis* complex

## Abstract

**Background:**

Red deer *(Cervus elaphus)* is regarded as an epidemiologically relevant host for *Mycobacterium bovis* (*M. bovis*) and closely related members of the *Mycobacterium tuberculosis* complex that cause animal tuberculosis (TB). The standard antemortem screening test for the detection of TB in deer is the intradermal tuberculin skin test, but the detection of interferon-gamma (IFNγ) produced by white blood cells exposed to *M. bovis* antigens can be used as an alternative or supplemental assay in most TB eradication/control programs. This study aims to develop an in-house sandwich ELISA for deer IFNγ, based on the cross-reactivity of the antibodies to both cervid and bovine IFNγ, and to evaluate the potential of this assay to detect *M. bovis*-infected red deer in response to the in vitro stimulation of whole-blood cells with bovine purified protein derivative (bPPD), p22 protein complex derived from bPPD or using the specific tuberculous mycobacterial proteins ESAT-6/CFP-10, Rv3615c and Rv3020c. The positive control stimulant used in this study was pokeweed mitogen, which resulted in a consistent induction of IFNγ in samples from red deer, thus allowing the interpretation of the assay.

**Results:**

The percentage of animals correctly classified by this technique as *M. bovis* non-infected was 100%. The detection of infected animals as positive was high and ranged widely depending upon the antigen and the cut-off value applied, as well as the time after infection. Our findings indicate that this protocol may serve as a reliable assay for the antemortem diagnosis of TB from the initial stage of *M. bovis*-infection, and may also be adequately sensitive.

**Conclusions:**

The suggested optimal antigens and cut-off are bPPD, p22 and the combination of ESAT-6/CFP-10 and Rv3020c with a 0.05 Δ optical density, which yielded a up to 100% correct classification of TB positive and negatve red deer under our experimental conditions. This technique will aid in TB testing of farmed and translocated deer. Future studies should evaluate the ability of this IFNγ assay to detect specific responses under field conditions.

**Electronic supplementary material:**

The online version of this article (10.1186/s12917-017-1262-6) contains supplementary material, which is available to authorized users.

## Background

Animal tuberculosis (TB) caused by *Mycobacterium bovis* (*M. bovis*) has been identified in a wide range of hosts, including both domestic animals and wildlife, which can become reservoirs of infection and contribute to infection maintenance [1]. This disease has a worldwide distribution and is still a major infectious disease among the domestic animals and wildlife in many countries [2–4]. The eradication of TB in cattle is based on diagnosing infection through the intradermal tuberculin test and the slaughter of infected animals or whole herds [5].

The red deer (*Cervus elaphus*) is considered an epidemiologically important wildlife host for *M. bovis* and closely related members of the *M. tuberculosis* complex (MTC) that cause TB [1]. Red deer are also hosts of *M. avium paratuberculosis,* which causes paratuberculosis (PTB) or Johne’s disease. These two diseases, TB and PTB, are a priority in the management and sanitary control of farmed deer [6–8]. Deer farming is a growing activity in Spain, since the animals produced are used to restock estates on which they are subsequently hunted [9]. Pre-movement tests are currently compulsory for the translocations of live deer in Spain.

Serological tests have been developed and employed to detect antibodies to *M. bovis* antigens in deer, but no assay has to date proven to have an adequate sensitivity (Se) or specificity (Sp) for routine TB diagnosis in deer [10–13]. The poor performance of this diagnostic approach can be attributed to the immunological response generated in ruminants infected with *M. bovis*, with a minor involvement of the humoral response which is initially counterbalanced by a more effective cell-mediated immune response (CMI) [14], widely recognized as the main factor involved in the containment of the infection [15]. TB diagnosis should, therefore, be based on the evaluation of CMI, which can be determined using the tuberculin skin test or, alternatively, interferon-gamma (IFNγ) release assays [16].

The standard antemortem screening test for TB detection in farmed deer is the intradermal tuberculin skin test, a diagnostic method designated by the World Organization for Animal Health [17] which is used worldwide. This test consists of the inoculation of purified protein derivative tuberculin (PPD) to measure the increase in the cervical skin fold thickness induced by a single inoculation of PPD prepared from *M. bovis* (bPPD) (single cervical skin-test, SCST). Another valid technique is the comparative cervical skin test (CCST), which consists of also injecting avian PPD, prepared from *M. avium* ssp. *avium* (aPPD) into a separate site in order to allow a comparative assessment of the skin fold increase [18, 19].

As an alternative or supplemental assay to the skin test, in most TB eradication/control programs the CMI response can also be measured in vitro by an assay that detects the IFNγ produced by peripheral blood mononuclear cells (PBMCs) exposed to *M. bovis* antigens [20, 21]. The IFNγ test has proven to be the best option, since this cytokine is thought to be involved in immunity to mycobacterial infections and is released in vitro, and thus readily measurable by enzyme-linked immunosorbent assays (ELISA) [20–22]. This assay has been accepted for use as an ancillary test to the intradermal test in the European Union since 2002 [Council Directive 64/ 432/EEC, amended by (EC) 1226/2002], as it provides national TB control programs and industry with an additional tool for use in curtailing the spread of TB in cattle and other *Bovidae* [16, 23]. The European Commission recently requested the European Food Safety Authority (EFSA) to issue a scientific opinion on the suitability of the IFNγ test for its inclusion in Directive 64/432/EEC as an official primary or stand-alone test and as an equivalent to the intradermal test employed to define the infectious status [24].

When compared to the skin test, the IFNγ avoids the continuous stimulation of the animal with PPDs, does not require the animal to be captured twice and prevents the technical variability associated with assessing skin test reactions [16, 23, 25]. In cattle, the IFNγ test has an increased Se with a slightly lower Sp than the intradermal tuberculin test [26, 27]. The Se and Sp of the IFNγ assay are estimated at 85 to 100% and 70 to 93%, respectively, with the use of bPPDs [23, 25, 27–29]. bPPDs consist of a complex mixture of proteins and include a great variety of antigens, many of which are shared with other mycobacterial species and closely related bacteria, something that may lead to a lack of Sp [30, 31]. Specific antigens, which are present in tuberculous mycobacteria and not in non-tuberculous mycobacteria or *M. bovis* Bacillus Calmette-Guérin (BCG), can be used for blood stimulation in the IFNγ assay in tests that discriminate between *M. avium*-exposed and BCG vaccinated individuals [28, 32–35], increasing the Sp of these diagnostic tests [25, 27–29, 36]. Several antigens have been described as potential diagnostic targets (e.g. ESAT-6/CFP-10, MPB70, MPB83, Rv3615c or Rv3020c) for skin testing, IFNγ assay or antibody response assessment in domestic livestock and wild animals [27, 36–40].

An assay was designed to detect the IFNγ produced by red deer leukocytes (Cervigam; Pfizer Animal Health) that also reacts with the IFNγ produced by reindeer *(Rangifer tarandus)*, white-tailed deer *(Odocoileus virginianus)* and other deer species leukocytes, indicating that antibodies within the assay are cross-reactive with the IFNγ produced in these different species [35, 41]. The in vitro measurement of IFNγ production, such as in the Cervigam assay, served as a useful test for the antemortem diagnosis of tuberculosis in Cervidae [41, 42]. However, this product is not currently commercially available, and it is therefore necessary to develop an alternative protocol suitable for cervids.

Since red deer are, from a taxonomic point of view, closely related to bovines, many of the tests developed for bovines used previously in cattle have also been described to work in deer. In particular, the high amino acid sequence homology between *Bos taurus* and *Cervus elaphus* IFNγ (91%, http://www.uniprot.org) made it likely that antibodies recognising bovine IFNγ would cross-react wtih cervid IFNγ. This study was designed to develop an in-house sandwich ELISA for the detection of cervid IFNγ in red deer, based on the cross-reactivity of the antibodies specific to bovine IFNγ, and to evaluate the potential of this assay to detect *M. bovis*-infected deer in response to the in vitro stimulation of whole-blood cells with bPPD, p22 protein complex derived from bPPD and aPPD or using specific *M. bovis* proteins, such as ESAT-6/CFP-10, Rv3615c or Rv3020c.

## Methods

### Animals and experimental design

Fifteen 7–8 month-old female red deer (*Cervus elaphus*) calves were obtained from a TB-free red deer farm in southern Spain (no positive cases and the use of CCST and bacterial isolation since 2003). All individuals were tested using ELISA to confirm their MTC antibody-free status before the study started. The animals were housed in a class III bio-containment at the NEIKER institute in Derio (Spain), where the calves were allowed to adapt for 1 week before starting the study and had ad libitum food and water.

An experimental infection with *M. bovis* was carried out as part of a vaccine trial [43]. For the challenge, a *M. bovis* field strain (2008/2575) was propagated in Middlebrook 7H9 broth containing 10% OADC Enrichment (Becton, Dickinson and Company, New Jersey, USA), 0.2% glycerol and 0.05% Tween 80 (*v*/v). Bacterial growth was harvested by means of centrifugation, washed twice and thoroughly re-suspended in phosphate-buffered saline (PBS) with 0.2% glycerol and 0.05% Tween 80. Bacterial concentration was calculated by plating serial dilutions on agar-solidified Middlebrook 7H9 and adjusted to 10^6^ colony forming units (CFU)/ml. The suspension was stored at −80 °C until used. The animals were sedated with xylazine (Xilagesic 2%; Laboratorios Calier, Barcelona, Spain) and the inoculum containing 10^6^ CFU of *M. bovis* in 10 ml of sterile PBS was administered by intratracheal injection with an 18 G needle.

Blood samples were collected at different time points during the experiment, including prior to the challenge (day 0), fifteen days post-inoculation (15 dpi), one month after challenge (30 dpi) and on the day of the necropsy (60 dpi).

The animals were sedated with xylazine (Xilagesic 2%) and euthanized by means of an intravenous injection of T-61 (Intervet S.A., Salamanca, Spain) at 60 dpi following the manufacturer’s instructions*.* All euthanized calves were subjected to a systematic necropsy with the main objective of assessing the presence and extension of tuberculous lesions. Samples were collected from head lymphoid tissues, including oropharyngeal tonsil and mandibular, parotid and retropharyngeal lymph nodes (LNs), lung, tracheobronchial and mediastinal LNs, spleen, ileocaecal valve and mesenteric and hepatic LNs. Liver, kidneys and LNs from other locations were sampled when suspicious lesions had been observed in these organs. The samples were placed individually in sterile bags and stored at −80 °C until the bacteriological culture took place.

### Microbiology

Samples of tissues for the culture of mycobacteria were thoroughly homogenized in sterile distilled water (2 g in 10 ml or equivalently). Five ml of this suspension were decontaminated and processed following the manufacturer’s instructions in order to inoculate BBL MGIT tubes supplemented with BBL MGIT PANTA and BACTEC MGIT growth supplement (Becton, Dickinson and Company, New Jersey, USA). BBL tubes were incubated for 42 days in a BACTEC MGIT 960 System. The remaining 5 ml were decontaminated in hexadecyl-pyridinium chloride at a final concentration of 0.75% (*w*/*v*) for 12–18 h. The samples were centrifuged at 2500 × g for 5 min and the pellets cultured in Coletsos (bioMèrieux, Madrid, Spain) and Lowenstein-Jensen with pyruvate (Difco, Becton, Dickinson and Company) tubes at 37 °C for 4 months. All isolates were spoligotyped in order to confirm the strain [44].

### Serum antibody detection

Sera obtained by centrifugation (3000 × g for 10 min) from blood samples at 0, 15, 30 and 60 dpi were tested in duplicate. We applied an in-house ELISA with some modifications was described previously by Boadella et al. [45], using bovine PPD (CZ Veterinaria SL, Lugo, Spain) as an antigen and protein G horseradish peroxidase (Sigma-Aldrich Química SA, Madrid, Spain) as a conjugate. Sample results were expressed as an ELISA percentage (E%) that was calculated using the following formula: [sample E% = (mean sample optical density (OD) / 2 × mean of negative control OD) × 100]. The cut-off values were defined as the ratio of the mean sample OD to the double of mean OD of the negative control. Serum samples with E% values greater than 100 were considered positive.

### IFNγ test

Blood samples were collected in tubes with lithium heparin at 0, 15, 30 and 60 dpi and processed at room temperature within the following 8 h. Blood samples were dispensed in aliquots into individual wells of a 24-well plate (Becton, Dickinson and Company, New Jersey, USA) with each antigen/control. Stimulation of whole blood was performed with aPPD (20 μg/ml), bPPD (20 μg/ml), a protein complex named p22 (20 μg/ml), and cocktails of synthetic peptides spanning the full sequences of early secretory antigenic target-6 kDa and culture filtrate protein 10 (ESAT-6/CFP-10; 5 μg/ml for each peptide; AHVLA), Rv3615c (5 μg/ml each peptide) and Rv3020c (5 μg/ml per peptide). The peptides cocktails were kindly provided by Drs G. Jones and M. Vordermeier (APHA) [46]. Pokeweed mitogen (PWM; 20 μg/ml; Sigma-Aldrich, Spain) was used as a positive control, while no stimulation (PBS) was employed as a negative control. The protocol was performed as described for other species [47, 48]. Blood cultures were incubated for 24 h at 37 °C in a humidified incubator (5% CO_2_). Plasma was harvested after 24 h and stored at −20 °C until assayed.

The IFNγ levels in non-stimulated and stimulated plasma were determined using an in-house sandwich ELISA that specifically detects this soluble cytokine using commercially available monoclonal antibody (mAbs) pairs for bovine IFNγ (Serotec, Oxford, UK). Microplates (Nunc Maxisorb, Roskilde, Denmark) were coated with 50 μl of highly purified anti-IFNγ Abs at 5 μg/ml in PBS (pH 7.5) and incubated at 4 °C overnight. After a blocking step with 75 μl of PBS, 0.05% Tween-20 and 2% bovine serum albumin for 1 h at room temperature, the plates were washed 3 times with PBS/Tween-20 and incubated with 50 μl of plasma diluted 1:2 for 1 h at 37 °C. The plates were then washed 3 times and incubated with 50 μl of biotinylated anti-IFNγ Abs at 5 μg/ml for 1 h at room temperature. This was followed by another washing step and the addition of 50 μl of streptavidin–peroxidase (1,2000; Southern Biotech, Birmingham, USA) for 45 min at room temperature. After a final wash, 100 μl of chromogenic substrate (Fast OPD, Sigma-Aldrich, Spain) were added and the reaction was stopped with 50 μl of 3 N H_2_SO_4_. The OD was measured in a spectrophotometer at 450 nm. Optimal concentrations of mAbs were determined by evaluating the responses to 1, 2, 5, 10 and 20 μg/ml of each Ab. Individual samples were analyzed in duplicate and the assay was repeated if the duplicate responses were not consistent. The data were presented as OD readings or as differences between the response to antigen or mitogen and the response to PBS (Δ OD). Results for a given sample were only validated when PWM Δ OD was higher than or equal to 0.5 and low OD readouts were obtained in non-stimulated wells (PBS wells).

IFNγ test was performed by an experienced researcher but with no previous knowledge of which sample was being analyzed. The interpretation of the responses was based on methods commonly used in the Spanish National Bovine Tuberculosis Eradication Program for Bovigam assay and in the former commercial Cervigam assay. The responses to the bPPD and p22 must exceed the responses to both aPPD and PBS by a given cut-off (0.1 or 0.05 Δ OD) to be considered positive. The responses to the specific antigens (ESAT-6/CFP-10, Rv3615c and Rv3020c) were compared with the responses to PBS.

### *M. bovis* proteins selection

bPPD consists of a complex mixture of proteins from *M. bovis* and includes a great variety of antigens, being the most common antigen used for in vitro IFNγ assays [27]. Recently, a new immunopurified subcomplex protein named as P22 was purified by affinity chromatography from bPPD (CZ Veterinaria SL, Porriño, Spain), and could become a solid alternative to bPPD for detecting antibodies against MTC. P22 is composed of several antigens including MPB83, ESAT-6, CFP-10 and, especially MPB70. It improves standardization and reproducibility by different laboratories (patent EP16382579).

ESAT-6 and CFP-10 are two of the major antigenic targets identified in both cattle and human MTC [49, 50]. These proteins are absent in BCG vaccine strains [51] and can therefore serve as targets for differentiating vaccinated from infected animals (DIVA test) [52]. The cocktail ESAT-6/CFP-10 supports the notion that a combination of epitopes of different antigens increases the diagnostic Se without compromising Sp [36, 53]. The antigen Rv3615c can also be used in combination with ESAT-6/CFP-10 (PC-HP, Prionics, Schlieren, Switzerland) or alone, detecting a significant portion of TB infected animals that were negative to the ESAT-6/CFP-10 peptides cocktail [54] or recognizing a significant proportion (37%) of infected animals but not in BCG vaccinates [39]. Moreover, Jones et al. demonstrated that the antigen Rv3020c also induced IFNγ production in whole blood from *M. bovis*-infected animals but not from BCG-vaccinated cattle [45]. Both candidate antigens have been tested for their DIVA capabilities and showed promising results when used as antigens in the BOVIGAM® assay as well as for skin testing [55–57].

### Statistical analyses

ELISA data were analyzed by means of a one-way variance analysis followed by a Tukey-Kramer multiple comparison test. This approach was followed to test for statistical differences in ELISA values through time. The t-test was employed to search for statistical differences in IFNγ levels (OD) in the different times post-inoculation with regard to 0 dpi for any of the mycobacterial antigens, mitogen and negative control used to stimulate IFNγ production. Pearson’s product-moment correlations were computed between the IFNγ levels in plasma stimulated with different antigens. Concordance between the IFNγ test and antibody levels to bPPD measured using ELISA was calculated by means of Cohen’s κ coefficient. Statistical analyses were run on GraphPad Prism 5 software (GraphPad Software, Inc., La Jolla, CA, USA).

## Results

The animals were observed daily throughout the experiment and clinical signs of TB were detected, including those of depression, cough, dyspnoea and open-mouth breathing. The intratracheal inoculation of *M. bovis* in the deer resulted in gross tuberculosis lesions in every animal, which were especially prominent in lung, tracheobronchial and mediastinal LNs. A bacteriological culture confirmed *M. bovis* infection in all the animals and spoligotyping confirmed the only presence of the administered *M. bovis* strain.

**Fig. 1:**
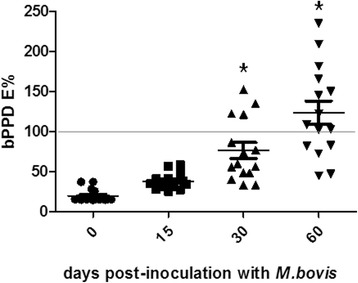
Individual red deer antibody levels (in ELISA percentage, E%) to bovine purified protein derivative (bPPD) measured by using ELISA immediately before and until 60 days post-inoculation of *M. bovis*. Serum samples with E% values greater than 100 were considered positive (horizontal grey line). *Significant differences (*p* < 0.05) between pre-inoculation values and those from different post-inoculation time points

ELISA responses to bPPD of infected deer were low before and immediately after (15dpi) infection but increased thereafter progresively up to 60 dpi (*p* < 0.05), when 70% of the infected animals were tested as positive to the disease with this technique (mean of 121%, with a cut-off equal to or greater than 100%) (Fig. 1 and Additional file 1. Table S1).

**Fig. 2:**
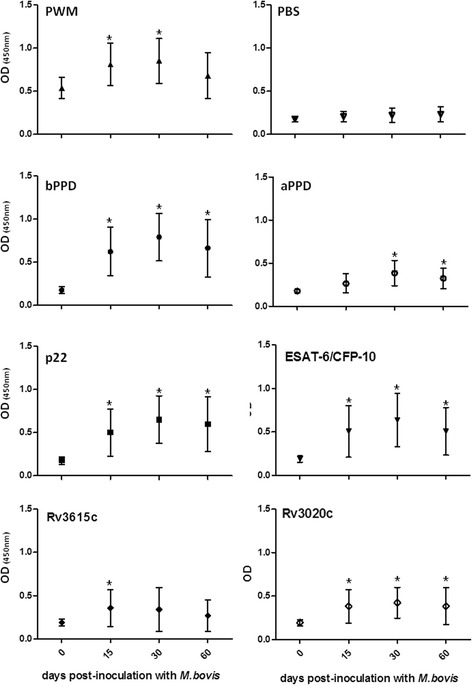
IFNγ responses in plasma from heparinized blood stimulated in *M. bovis*-infected red deer (*n* = 15) presented as mean (±SD) of the optical density at 450 nm (OD_450nm_) to Pokeweed mitogen (PWM), negative control (phosphate-buffered saline, PBS), bovine and avian purified protein derivatives (PPD), p22; early secretory antigenic target-6 kDa and culture filtrate protein 10 (ESAT-6/CFP-10), Rv3615c and Rv3020c. *Statistically significant differences (p < 0.05) observed at specific time points since *M. bovis* infection in comparison to pre-inoculation values

The stimulation of whole-blood cultures with PWM after 24 h of incubation at 37 °C led to responses of IFNγ in plasma that exceeded an OD of 0.5 at every time point tested. The production of IFNγ increased significantly during the course of the study (0.5 at the time of inoculation when compared to 0.88 at 30 dpi; *p* < 0.05) (Fig. 2), but some of the animals became less responsive to PWM (PWM OD < 0.5) at 60 dpi. However, controls (PBS) were consistently low throughout the experiment (OD, 0.20 ± 0.018), and we found no alterations after infecting the deer with *M. bovis* (Fig. 2 and Additional file 2. Table S2).

As early as 15 dpi, and at most time points thereafter until the completion of the study (60 dpi), the IFNγ responses of the infected deer to bPPD and p22 significantly exceeded those prior to the challenge (*p* < 0.05), peaking at 30 dpi (0.88 for bPPD and 0.67 for p22; *p* < 0.001). Several specific antigens produced by pathogenic mycobacteria of the *M. tuberculosis* complex but not by environmental or non-*M. tuberculosis* complex pathogens were evaluated as an approach to decrease the detection likelihood of false positive animals. We observed that whole blood samples stimulated with ESAT-6/CFP-10 and Rv3020c significantly increased the levels of IFNγ after *M. bovis* challenge during the course of the experiment (*p* < 0.05). This was not the case for Rv3615c, which induced signficantly elevated responses only at 15 dpi (*p* < 0.05;Fig. 2). The ESAT-6/CFP-10 and Rv3020c responses followed kinetics similar to those shown by PPDs and PWM, peaking at 30 dpi and dropping slightly at 60 dpi. The responses of IFNγ to aPPD differed at 30 and 60 dpi, but only increased by 0.2 in OD when compared with the pre-infection levels (Fig. 2).

The mean IFNγ responses (averaged over various time points) of infected deer to different mycobacterial antigens with regard to PBS in whole-blood were also evaluated. At the time of infection, the OD values for mycobacterial antigen stimulation were very low and similar to those of PBS. There were no significant differences at this time, with the exception of IFNγ levels in response to PWM (*p *< 0.05). The responses in samples stimulated with aPPD, Rv3615c and Rv3020c remained low during the course of the study and were not statistically different from PBS (*p* < 0.05) after infection, with the exception of an increase to Rv3020c at 30 dpi. However, the IFNγ responses of infected deer were higher at all time points after stimulation with either PWM, bPPD, p22 and ESAT-6/CFP-10 as compared to those with PBS, and there were statistically significant changes in all the antigens tested (*p* < 0.001). The mean IFNγ responses decreased only slightly at 60 days after *M. bovis* infection.

The interpretation of the responses to different antigens was based on protocols commonly used for the Bovigam assay in cattle or other farmed bovines. The responses to bPPD and p22 must be higher to both aPPD and PBS responses by 0.1 or 0.05 ΔOD to be considered positive. The responses to the specific antigens (ESAT-6/CFP-10, Rv3615c and Rv3020c) were compared to PBS (OD > 0.05 or 0.1). In order to evaluate the nonspecific responses to *M. bovis* antigens in non-infected deer, samples were collected from the TB-free deer before *M. bovis* inoculation (*n* = 15) and the production of IFNγ in response to mycobacterial antigens and mitogen was measured. All deer had responses to PWM (OD > 0.5); however, none of them had a response to mycobacterial antigens, independently of the cut-off value (data not shown).

Only results in which the control mitogen responses exceeded OD values of 0.5 were used for the evaluation of the test. Only 10 animals had PWM responses that exceeded 0.5 ΔOD at 60 dpi. Table 1 shows the differences in the percentage of positive samples to the IFNγ assay regarding *M. bovis*-infected deer depending on the time post-infection, the antigen used or the cut-off value. The differences in test Se were therefore detected at different times of infection for every mycobacterial antigen analyzed with both cut-off points. We observed a higher proportion of positive samples at 30 dpi, which decreased considerably at necropsy time (60 dpi), when the number of animals with a response to the mitogen also declined. In the earlier stages of infection, a higher proportion of positive samples was obtained with bPPD, p22 and ESAT-6/CFP-10 (85% to 92.8% Se at 15 dpi and 85% to 100% Se at 30 dpi) when compared to Rv3615c and Rv3020c (40% to 66.6% Se at 15 dpi and 26.6% to 100% Se at 30 dpi). The specific antigens ESAT-6/CFP-10, Rv3615c and Rv3020c are usually applied in parallel, and a test is thus positive if an animal responds to one of these cocktails. The evaluation in parallel of ESAT-6/CFP-10 with Rv3615c and Rv3020c considerably increased the Se of the technique when compared to the separate use of these antigens. At a cut-off value of 0.1 there were more differences between antigens but the detection of animals infected with *M. bovis* by the test was lower, whereas smaller differences and a higher percentage of infected animals were found when using 0.05 as the cut-off for the assay (Table 1).

**Table 1:** Percentage (%) of positive samples to IFNγ assay (95% Wilson’s confidence intervals) from *M. bovis*-infected deer

	15 dpi		30 dpi		60 dpi	
	< 0.1 (%)	< 0.05 (%)	< 0.1 (%)	< 0.05 (%)	< 0.1 (%)	< 0.05 (%)
bPPD	92.8 (68.5–98.7)	92.8 (68.5–98.7)	100 (79.6–100)	100 (79.6–100)	75 (40.9–92.9)	75 (40.9–92.9)
P22	85.7 (60.1–96)	92.8 (68.5–98.7)	100 (79.6–100)	100 (79.6–100)	75 (40.9–92.9)	87.5 (52.9–97.8)
ESAT-6/CFP-10	92.3 (66.7–98.6)	92.3 (66.7–98.6)	85.7 (60.1–96)	100 (79.6–100)	75 (40.9–92.9)	75 (40.9–92.9)
Rv3615c	40 (19.8–64.3)	40 (19.8–64.3)	26.6 (10.9–52)	26.6 (10.9–52)	25 (7.1–59.1)	37.5 (13.7–69.4)
Rv3020c	53.3 (41.7–84.8)	66.6 (30.1–75.2)	73.3 (48–89.1)	100 (79.6–100)	75 (40.9–92.9)	87.5 (52.9–97.8)
ESAT-6/CFP-10 + Rv3615c	92.3 (66.7–98.6)	92.3 (66.7–98.6)	86.6 (62.1–96.3)	100 (79.6–100)	75 (40.9–92.9)	87.5 (52.9–97.8)
ESAT-6/CFP-10 + Rv3020c	93.3 (70.2–98.8)	93.3 (70.2–98.8)	100 (79.6–100)	100 (79.6–100)	75 (40.9–92.9)	87.5 (52.9–97.8)

*IFNγ* interferon gamma, *bPPD* bovine purified protein derivative, *ESAT-6/CFP-10* early secretory antigenic target-6 kDa and culture filtrate protein 10; dpi: days post-infection

IFNγ responses to PWM, bPPD, aPPD, p22 and ESAT-6/CFP-10 were strongly correlated in *M. bovis*-infected deer (*p* < 0.001). The correlations between the responses to Rv3615c, Rv3020c and the other mycobacterial antigens were moderately positive (*p* < 0.05) (Additional file 3. Table S3).

The agreement (*kappa* value) reported between the IFN-γ assays and the antibody levels to bPPD measured using ELISA depended on the time post-infection, the antigen used and the cut-off value applied (Additional file 4. Table S4). The agreement between these techniques was 0 at the early stage of the disease, regardless of the cut-off value applied. Thereafter, the agreement between both techniques increased for all the antigens when micobacterial antibodies were produced, except in the case of Rv3615c. At necropsy time, the agreement obtained between the IFN-γ assay and ELISA for bPPD antibodies analyzed with the cut-off detailed in Spanish guidelines (0.05) varied from good to very good (0.73 < *k* < 0.86) in the case of all the antigens used, with the exception of Rv3615c, and good values of proportion of positive samples were maintained with this IFN-γ test (Table 1).

## Discussion

The main goal of this study was to assess the ability of an in-house sandwich ELISA to detect the IFNγ produced by *M. bovis*-infected deer in response to in vitro stimulation with bPPD, p22 and aPPD or using specific *M. bovis* proteins, such as ESAT-6/CFP-10, Rv3615c or Rv3020c.

The development of IFNγ assays is an important advance in the diagnosis of TB infection as regards measuring an effective CMI response. The results of our study also demonstrate that the in-house sandwich ELISA based on the cross-reactivity with bovine IFNγ detects the IFNγ response produced by stimulated leukocytes from experimentally *M. bovis*-infected red deer. The optimization of an in-house sandwich ELISA has many advantages because it is relatively inexpensive and can be easily automated to process large numbers of samples. Moreover, the ability to modify IFNγ test parameters provides challenges to ensure the standardization of testing procedures and quality assurance, making it possible to provide a closer adaptation of this assay to the needs of different species of wild ruminants within a TB program.

The initial development of IFNγ-based tests for TB surveillance requires a powerful stimulation with mitogens or superantigens to effectively demonstrate the functional capacity of the sample or to detect an underlying CMI response suppression, thereby reducing the risk of false-negative test results. Prior studies have identified the PWM as a reliable trigger of the IFNγ production in other cervids [11, 41, 42, 58]. PWM was also the mitogen used as the positive control stimulant in this study and resulted in a consistent induction of acceptable quantities of IFNγ in samples from red deer, thus favoring the interpretation of the assay. Although PWM elicited a consistent positive control stimulus for the leukocytes with an OD greater than or equal to 0.5, the highest OD observed was 0.88, while in other experiences with reindeer the OD of the IFNγ response fluctuated from 1 to 1.8 when using the Cervigam test [41, 42, 58]. However, Waters et al. (2008) found a low OD response to the mitogen and evidenced of a limited usefulness of the Cervigam in samples from white-tailed deer, elk (*Cervus canadensis*) and fallow deer (*Dama dama*) [58].

The red deer used in this study were obtained from a TB-free farm and were negative to antibodies against bPPD when using ELISA before the experiment started. The animals were intratracheally challenged with *M. bovis* and infection was confirmed by means of pathology and culture. It is worth noting that some red deer responded with antibodies to bPPD from 30 dpi onwards and that, according to the ELISA test, most of them were positive to *M. bovis* at necropsy time (60 dpi). The antibody response was not directly correlated with the IFNγ results until 60 dpi (*k* = 0.73 with 95% confidence intervals), given that the development of humoral immunity to *M. bovis* takes longer than that of cell-mediated immunity, even in deer where the humoral immune response is initiated earlier than in other animals [6].

In effective TB eradication programs, the early detection of infected animals is a key to avoid maintaining and disseminating the infection. Significant efforts have therefore been made to improve the Se of diagnostic methods using ancillary tests such as the IFNγ assay. This test is expected to identify infected individuals at an earlier stage of infection than the skin test and the antibody ELISA [16, 59–62]. In this study, IFNγ production was found as early as 15 dpi, thus enabling us to diagnose the disease at the initial stage, prior to the detection of serum antibodies.

Both nonspecific responses to PWM and specific responses to mycobacterial antigens varied widely between individual deer over time. This led us to exclude some samples at necropsy time owing to their inability to produce IFNγ in response to PWM (OD less than 0.5). This poor response to mitogen stimulation indicated a concurrent poor response to mycobacterial antigens, suggesting that either the assay was unable to detect the cytokine consistently or the leukocytes from *M. bovis*-infected deer were unable to produce IFNγ after 60 dpi. The inconsistent detection of IFNγ in the assay may be discarded because we detected a good response of this cytokine throughout the study with the protocol developed; the alterations in the immune response that affect the kinetics of the cytokine response would, therefore, be the most probably associated factor. Some immunological factors that depress the cell-mediated immunuty decrease the probability of detecting infected cattle in a context of impaired CMI response to mycobacterial antigens in IFNγ and skin tests, thus affecting their Se [58, 63, 64]. This state of “anergy” is recorded in animals subjected to stress and in cattle with advanced or generalized TB [10, 65]. This agrees with the findings observed in our study, in which the deer inoculated intratracheally with 10^6^ CFU of *M. bovis* had generalized TB with a massive growth of mycobacteria in different tissues [43], which could explain this state of immunosuppression at 60 dpi. However, the suppression of the CMI appears to drift to a humoral immune response according to the response of antibodies to bPPD observed at necropsy time. This suggests that a combination of CMI-based techniques and the ELISA as an ancillary test for the detection of antibodies may increase the detection of infected animals, thus helping to control TB [66].

bPPD and aPPD, along with antigens specific for *M. bovis,* have been evaluated in this study for the diagnosis of TB in red deer using the IFNγ test. Responses to bPPD and p22 greater than 0.1 or 0.05 ΔOD with regard to aPPD and PBS are generally considered positive, while the responses to ESAT-6/CFP-10, Rv3615c and Rv3020c were compared only with PBS. However, cut-off levels similar to those for tuberculin are being applied in cattle when considering these antigens (antigen minus PBS > 0.05 or 0.1) [45]. In the current study, we used samples obtained before the challenge and no deer had an IFNγ response to any of the antigens tested, as in previous studies of ruminants [25, 28, 29, 67]. After infection, the highest percentage of detection of infected deer with the IFNγ assay was for bPPD at the initial stage of the disease (92.8 to 100%) in comparison with other antigens, which is again in parallel with previous reports for ruminants [23, 25, 28, 29, 36, 41]. These results were the same as those obtained with the p22 stimulation when the 0.05 threshold was used (*r* = 0.9; *p* < 0.0001). However, slight differences in the identification of tuberculous animals were detected in the positive samples when using different antigens and the 0.1 cut-off point, indicating a lower degree of variation with a more stringent cut-off.

In this study, TB gave rise to a very slight cross-reactivity with aPPD, since OD values of aPPD were considerably lower than the readings produced when using bPPD. In field conditions, the exposure to *M. avium* or environmental non-tuberculous *Mycobacterium spp*. may induce cross-reactive responses with *M. bovis* antigens [68]. The presence of PTB or other infections caused by environmental mycobacteria is an interference factor in the diagnosis of TB using PPDs [67], but not using the ESAT-6/CFP-10 protein cocktail [68]. A limitation of this study was not including samples from animals infected with other species of mycobacteria as control owing to limited BSL3 laboratory space availability. Further studies are necessary to estimate the effects of these other mycobacteria, in addition to the single and comparative skin test, on IFNγ responses and to evaluate the mechanisms involved in these effects.

We compared the potential of specific antigens, present only in tuberculous mycobacteria and absent in non-tuberculous mycobacteria (ESAT-6/CFP-10, Rv3615c and Rv3020c), for their use in this diagnostic test. In particular, the use of these antigens may enhance the Sp of IFNγ-based tests in comparison to that achieved when standard PPDs are used as the eliciting agent.

The percentage of *M. bovis*-infected deer postitive to the technique with individual *M. bovis* specific antigens ranged widely depending on the antigens and the cut-off value applied. Proteins encoded within the ESAT-6 gene cluster (including ESAT-6 and CFP-10) of tuberculous mycobacteria play an important role in the pathogenesis of the disease, inducing potent T-cell responses that have been used in TB diagnostic tests [28, 69, 70]. In the present study, the peptides cocktail combining ESAT-6 and CFP-10 elicited robust recall IFNγ responses in *M. bovis*-infected deer from 15 dpi onwards and reached a satisfactory detection of infected animals (92.3 to 100%), which is higher than in previous studies of domestic ruminants [28, 36, 39, 53, 65]. However, the relatively small sample size in our experiment resulted in a wide 95% CI, and future studies are required to confirm this observation under field conditions. It is possible, for example, that the high detection of *M. bovis*-infected animals seen in our study could be owing to the fact that all the animals used were exposed to a high dose of *M. bovis* and the samples were collected at an early stage of the disease. This may have led us to observe a decrease in the detection of TB-positive animals with all the antigens at 60 dpi due to a possible anergy of the animals.

In contrast to the responses to ESAT-6/CFP-10, the responses of *M. bovis-*infected deer to Rv3615c only slightly exceeded the pre-infection values, with a maximum detection of TB-positive deer at 15 dpi when 40% of the animals elicited an IFNγ response to the stimulation, similar to that which has been previously reported for cattle [39]. Various authors have reported that the use of Rv3615c in combination with ESAT-6 and CFP10 appears to increase the detection of TB-positive deer without false positives in the IFNγ assay [39, 56]. This observation is suggested by our results, were the use of antigen combinations provided better detection of infected animals. In particular, Rv3615c or Rv3020c were found to recognize infected deer missed by ESAT-6/CFP-10.

## Conclusions

These findings indicate that the in-house sandwich ELISA developed for red deer IFNγ and based on the cross-reactivity with bovine IFNγ may serve as a valuable assay for the antemortem diagnosis of TB in deer experimentally infected with *M. bovis*, as early as 15 dpi. The suggested optimal antigens and cut-off are bPPD, p22 and the combination of ESAT-6/CFP-10 and Rv3020c with a 0.05 ΔOD. This yielded the detection of up to 100% of the positive and negatve deer under our experimental conditions. This technique will aid in TB testing of farmed and translocated deer. It would be useful to carry out further studies in order to evaluate the ability of this IFNγ assay to detect specific responses in field conditions.

## Additional files

Additional file 1: Table S1. Individual antibody levels (in ELISA percentage, E%) to bovine purified protein derivative (bPPD) in red deer immediately before and until 60 days post-inoculation of *M. bovis*. Sera with E% values greater than 100 were considered positive. (XLS 25 kb)

Additional file 2: Table S2. Individual optical densities at 450 nm reflecting IFNγ responses in plasma of *M. bovis*-infected red deer from heparinized blood stimulated with Pokeweed mitogen (PWM), negative control (phosphate-buffered saline, PBS), bovine and avian purified protein derivatives (PPD), p22, early secretory antigenic target-6 kDa and culture filtrate protein 10 (ESAT-6/CFP-10), Rv3615c and Rv3020c. (XLS 33 kb)

Additional file 3: Table S3. Correlations between the IFNγ responses (optical density) of whole-blood stimulated with a mitogen (PWM), not stimulated (PBS) and with mycobacterial antigens from *M. bovis*-infected deer. (DOCX 15 kb)

Additional file 4: Table S4. Test agreement results - Kappa (κ) values with 95% confidence intervals (CI_95_) between the evaluated assays in *M. bovis*-infected deer. (DOCX 16 kb)

## Abbreviations

aPPD: Avian PPD
BCG: Bacillus Calmette-Guérin
bPPD: Bovine PPD
CCST: Comparative cervical skin test
CFU: Colony forming units
CMI: Cell-mediated immune response
dpi: Days post-infection
E%: ELISA percentage
EFSA: European Food Safety Authority
ELISA: Enzyme-linked immunosorbent assays
ESAT-6/CFP-10: Early secretory antigenic target-6 kDa and culture filtrate protein 10
IFNγ: Interferon gamma
LNs: Lymph nodes
*M. bovis*: *Mycobacterium bovis*
mAbs: Monoclonal antibodies
MTC: *M. tuberculosis* complex
OD: Optical density
p22: Protein complex purified bPPD tuberculin
PBMCs: Peripheral blood mononuclear cells
PBS: Phosphate-buffered saline
PPD: Purified protein derivative
PTB: Paratuberculosis
PWM: Pokeweed mitogen
SCST: Single cervical skin-test
Se: Sensitivity
Sp: Specificity
TB: Tuberculosis
