# Supplementary material for: Development and evaluation of an interferon gamma assay for the diagnosis of tuberculosis in red deer experimentally infected with Mycobacterium bovis
Source: BMC Vet Res. 2017 Nov 16;13:341. doi: 10.1186/s12917-017-1262-6 (PMC5691593; doi:10.1186/s12917-017-1262-6)
Supplement: Supplementary file 3 — Correlations between the IFNγ responses (optical density) of whole-blood stimulated with a mitogen (PWM), not stimulated (PBS) and with mycobacterial antigens from M. bovis-infected deer. (DOCX 15 kb) [file 12917_2017_1262_MOESM3_ESM.docx]

| **Additional file 3: Table S3** Correlations between the IFNγ responses (optical density) of whole-blood stimulated with a mitogen (PWM), not stimulated (PBS) and mycobacterial antigens from *M. bovis*-infected deer. | | | | | | | | | | | | | | | |
| --- | --- | --- | --- | --- | --- | --- | --- | --- | --- | --- | --- | --- | --- | --- | --- |
|  | PWM |  | bPPD |  | aPPD |  | p22 |  | ESAT-6/CFP-10 |  | Rv3615c |  | Rv 3020c |  | PBS |
| PWM | - |  | 0.8 |  | 0.7 |  | 0.8 |  | 0.7 |  | 0.5 |  | 0.5 |  | 0.2 |
| bPPD |  |  | - |  | 0.8 |  | 0.9 |  | 0.7 |  | 0.5 |  | 0.5 |  | 0.2 |
| aPPD |  |  |  |  | - |  | 0.8 |  | 0.8 |  | 0.4 |  | 0.4 |  | 0.3 |
| p22 |  |  |  |  |  |  | - |  | 0.8 |  | 0.5 |  | 0.5 |  | 0.2 |
| ESAT-6/CFP-10 |  |  |  |  |  |  |  |  | - |  | 0.4 |  | 0.5 |  | 0.4 |
| Rv3615c |  |  |  |  |  |  |  |  |  |  | - |  | 0.5 |  | 0.4 |
| Rv 3020c |  |  |  |  |  |  |  |  |  |  |  |  | - |  | 0.5 |
| PBS |  |  |  |  |  |  |  |  |  |  |  |  |  |  | - |
| IFNγ: interferon gamma; PWM: Pokeweed mitogen; bPPD: bovine purified protein derivative; aPPD: avian PPD; ESAT-6/CFP-10: early secretory antigenic target-6kDa and culture filtrate protein 10; PBS: phosphate-buffered saline | | | | | | | | | | | | | | | |
|  |  |  |  |  |  |  |  |  |  |  |  |  |  |  |  |
