# Supplementary material for: Development and evaluation of an interferon gamma assay for the diagnosis of tuberculosis in red deer experimentally infected with Mycobacterium bovis
Source: BMC Vet Res. 2017 Nov 16;13:341. doi: 10.1186/s12917-017-1262-6 (PMC5691593; doi:10.1186/s12917-017-1262-6)
Supplement: Supplementary file 4 — Test agreement results - Kappa (κ) values with 95% confidence intervals (CI95) between the evaluated assays in M. bovis-infected deer. (DOCX 16 kb) [file 12917_2017_1262_MOESM4_ESM.docx]

| **Additional file 4: Table S4** Test agreement results - Kappa (κ) values with 95% confidence intervals (CI_95_) between the evaluated assays in *M. bovis*-infected deer (n =15). | | | | | | |  |  |
| --- | --- | --- | --- | --- | --- | --- | --- | --- |
|  |  | **15 dpi** |  | **30 dpi** |  | **60 dpi** | |  |
| *IFNγ test* |  | *Antibody levels to bPPD measured using ELISA* | | | | |  |  |
|  |  |  |  |  |  |  | |  |
| bPPD^a^ |  | 0 |  | 0 |  | 0.73 | |  |
| bPPD^b^ |  | 0 |  | 0 |  | 0.73 | |  |
| aPPD^a^ |  | 0 |  | 0.30 |  | 0.60 | |  |
| aPPD^b^ |  | 0 |  | 0.33 |  | 0.73 | |  |
| p22^a^ |  | 0 |  | 0 |  | 0.73 | |  |
| p22^b^ |  | 0 |  | 0 |  | 0.86 | |  |
| ESAT-6/CFP-10^a^ |  | 0 |  | 0.10 |  | 0.73 | |  |
| ESAT-6/CFP-10^b^ |  | 0 |  | 0 |  | 0.86 | |  |
| Rv3615c^a^ |  | 0 |  | 1 |  | 0.23 | |  |
| Rv3615c^b^ |  | 0 |  | 1 |  | 0.34 | |  |
| Rv 3020c^a^ |  | 0 |  | 0.23 |  | 0.73 | |  |
| Rv 3020c^b^ |  | 0 |  | 0 |  | 0.86 | |  |
| ^a^0.1 cut-off point; ^b^0.05 cut-off point; IFNγ: gamma interferon; bPPD: bovine purified protein derivative; aPPD: avian PPD; ESAT-6/CFP-10: early secretory antigenic target-6kDa and culture filtrate protein 10; dpi: days post-infection. | | | | | | | |  |
|  |  |  |  |  |  |  | |  |
|  | | | | | | |  |  |
